# Molecular effects of cardiac contractility modulation in patients with heart failure of ischemic aetiology uncovered by transcriptome analysis

**DOI:** 10.3389/fcvm.2024.1321005

**Published:** 2024-02-01

**Authors:** E. Lyasnikova, K. Sukhareva, M. Vander, K. Zaitsev, M. Firulyova, A. Sergushichev, M. Sorokina, M. Trukshina, V. Galenko, T. Lelyavina, L. Mitrofanova, K. Simonova, M. Abramov, G. Faggian, G. B. Luciani, D. S. Lebedev, E. N. Mikhaylov, M. Sitnikova, A. Kostareva

**Affiliations:** ^1^ Institute of Molecular Biology and Genetics, Almazov National Medical Research Centre, Saint Petersburg, Russia; ^2^ Graduate School of Life and Health Science, University of Verona, Verona, Italy; ^3^ Computer Technologies Laboratory, ITMO University, Saint Petersburg; ^4^ Department of Women’s and Children’s Health and Center for Molecular Medicine, Karolinska Institute, Stockholm, Sweden

**Keywords:** CCM, autophagy, optimizer, heart failure, sarcomeric genes, RNA sequencing

## Abstract

Cardiac contractility modulation (CCM) is based on electrical stimulation of the heart without alteration of action potential and mechanical activation, the data on its fundamental molecular mechanisms are limited. Here we demonstrate clinical and physiological effect of 12 months CCM in 29 patients along with transcriptomic molecular data. Based on the CCM effect the patients were divided into two groups: responders (*n* = 13) and non-responders (*n* = 16). RNA-seq data were collected for 6 patients before and after CCM including 3 responders and 3 non-responders. The overall effect of CCM on gene expression was mainly provided by samples from the responder group and included the upregulation of the genes involved in the maintenance of proteostasis and mitochondrial structure and function. Using pathway enrichment analysis, we found that baseline myocardial tissue samples from responder group were characterized by upregulation of mitochondrial matrix-related genes, Z disc-protein encoding genes and muscle contraction-related genes. In summary, twelve months of ССM led to changes in signaling pathways associated with cellular respiration, apoptosis, and autophagy. The pattern of myocardial remodeling after CCM is associated with initial expression level of myocardial contractile proteins, adaptation reserves associated with mitochondria and low expression level of inflammatory molecules.

## Introduction

Cardiac contractility modulation (CCM) has been actively studied in order to improve the prognosis for patients with heart failure and reduced ejection fraction (HFrEF) (1–3)_._ CCM is relatively new method of electrophysiological therapy for patients with HFrEF which is based on electrical stimulation of the heart in an absolute refractory period (1). CCM does not lead to the emergence of an action potential and does not change the course of electrical and mechanical activation of the heart, but only provides a positive inotropic effect without increasing the myocardial oxygen demand (4–6). The molecular mechanisms underlying the positive inotropic effect of CCM are suggested to be mediated by the increase in intracellular calcium levels and changes in phosphorylation of the key proteins modulating the activity of sarcoplasmic calcium (3–7). Until now, the only study focused on transcriptomic and proteomic changes measured directly in human cardiac tissue under CCM has been published (8). This study included only a target analysis of genes involved in calcium metabolism after tree and six months of 3-month long CCM. The data on the global molecular events in myocardial tissue under long-term exposure to CCM are still missing. The present study aimed to identify differentially expressed genes in myocardium biopsies obtained from patients with HFrEF after twelve months of CCM using whole-transcriptome sequencing approach in order to associate these alterations with a type of CCM response and myocardial remodeling.

## Materials and methods

The study protocol was approved by the local ethics committee of the Almazov National Medical Research Centre and complied with the requirements of the Declaration of Helsinki. All patients signed written informed consent. Patient characteristics and therapy are presented in Table 1.

**Table 1:** Demographic characteristics and clinical course of 6 patients with repeated endomyocardial biopsy samples, mean ± SD or median [Q25; Q75].

	Responders (*n* = 3)			Non-responders (*n* = 3)		
Age (years)	57.0 ± 9.0			47.7 ± 7.0		
Male/female	1/2			3/0		
	Baseline	6 months	12 months	Baseline	6 months	12 months
HR, beats/min	63.3 ± 3.1	66.3 ± 6.5	64.4 ± 15.1	67.0 ± 4.0	70.7 ± 6.8	76.7 ± 11.9
SBP, mmHg	110.0 ± 10.0	110.0 ± 07	110.0 ± 10.0	103.3 ± 5.8	116.7 ± 5.8	110.0 ± 17.3
QRS, ms	129.2 ± 4.0	133.0 ± 12.0	137.0 ± 14.4	113.7 ± 13.3	113.9 ± 10.8	111.0 ± 12.3
6MWT, m	391.3 ± 137.8	396.6 ± 59.2	416.7 ± 57.9	423.7 ± 105.6	453.7 ± 76.5	454.0 ± 79.9
Eq5D (visual analog scale), %	76.7 ± 20.8	88.3 ± 16.0	91.7 ± 7.6	63.3 ± 37.9	83.3 ± 5.6	61.7 ± 27.5
CCM-stimulation, %	98 [98;99]	99 [98;99]	93 [56;99]	98 [98;98]	98 [67;99]	97 [92;98]

HR, heart rate; SBP, systolic blood pressure; 6MWT, six-minute walk test.

Total RNAs were isolated from myocardial biopsy specimens obtained from right ventricular side of interventricular septum and libraries for RNA sequencing were prepared using TruSeq Stranded mRNA kit (Illumina, USA). We used DeSeq2 to perform differential expression analysis and compared patients before CCM implantation against patients after implantation. Raw sequencing data are available at SRA, NCBI, under GSE251971. Details of RNA sequencing and bioinformatic analysis are described in Suppl. File.

## Results

The mean follow-up period for all 29 patients with HFrEF of ischemic aetiology after CCM intervention was 11.8 ± 1.5 months, by the end of the first year, 11 patients (38%) had an ICD implanted mainly as a primary prevention of sudden cardiac death. After 6 and 12 months of CCM, a reduction in the NYHA (New York Heart Association) class accompanied by a significant improvement of echocardiographic, clinical, biochemical and quality of life parameters were reported (Supplementary Table S1). Based on the CCM effect on LVESV (Left Ventricular End-Systolic Volume), the study cohort was divided into two groups: responders (*n* = 13), where LVESV decreased by more than 10% compared to baseline after 6 months of CCM, and non-responders (*n* = 16), where parameters remained unaltered. These groups did not differ significantly in age, baseline hemodynamic and clinical parameters (Table 2). The degree of left ventricular (LV) reverse remodeling was significantly greater in the responder group compared to the non-responder group (Table 2). Both groups showed a significant reduction in the NYHA class, an increase in the distance 6MWT (6 Minute Walk Test) and quality of life according to EQ-5D data.

**Table 2:** Comparison of baseline characteristics and clinical course among the responder and non-responder groups (*n* = 29). Data presented as: *n* (%), mean ± SD or Me [Q25; Q75].

	Responders (*n* = 13)			Non-responders (*n* = 16)		
Age (years)	57.7 ± 6.5			54.0 ± 10.9		
Male /female	11 (85%)/2 (15%)			14 (87%)/2 (13%)		
ICD before CCM implantation	1 (8%)			4 (25%)		
Clinical course after CCM implantation						
ICD insertion after CCM implantation	6 (46%)			5 (31%)		
Device pocket stimulation, *n* (%)	1 (8%)			2 (12.5%)		
Leads replacement (2 RV leads), *n* (%)	1 (8%)			2 (12.5%)		
HF hospitalization, *n* (%)	–			2 (12.5%)		
Clinical status						
	Baseline	6 months	12 months	Baseline	6 months	12 months
NYHA FC, *n* (%)						
I	0	0 (0%)	1 (8%)	0 (0%)	3 (19%)	2 (13%)
II	9 (69%)	13 (100%)	12 (92%)	12 (75%)	13 (81%)	13 (81%)
III	4 (31%)	0 (0%)	0 (0%)	4 (25%)	0 (0%)	1 (6%)
NYHA FC						
Me [Q25; Q75],	2[2; 3]	2[2; 2]	**2[2; 2]** *	2[2;2.5]	**2[2; 2]** *	**2[2; 2]** *
mean ± SD**	2.3 ± 0.5	2.2 ± 0	1.9 ± 0.3	2.3 ± 0.4	1.8 ± 0.4	1.9 ± 0.4
HR, beats/min	68.0 ± 6.8	66.6 ± 8.3	63.2 ± 8.3	66.3 ± 7.2	65.4 ± 5.8	66.9 ± 9.9
SBP, mmHg	115.7 ± 16.3	115.7 ± 12.8	113.2 ± 12.9	114.8 ± 8.0	118.4 ± 6.5	120.0 ± 14.7
QRS, ms	115.9 ± 18.4	117.8 ± 21.9	121.4 ± 22.1	114.8 ± 18.8	115.8 ± 19.1	118.8 ± 21.5
6MWT, m	386.6 ± 70.4	**417.6 ± 46.3** **(*p* = 0.043)**	**446.9 ± 59.4** **(*p* = 0.01)**	375.3 ± 89.3	**410.4 ± 88.7** **(*p* = 0.041)**	**457.6 ± 86.6** **(*p* = 0.0002)**
LVEF, %	24.9 ± 6.1	**31.5 ± 6.4** **(*p* = 0.004)**	**30.7 ± 6.8** **(*p* = 0.01)**	26.4 ± 5.5	26.4 ± 6.6	27.0 ± 6.4
LV end-diastolic volume, ml	251.5 ± 57.9	**210.4 ± 37.1** **(*p* = 0.0003)**	**222.1 ± 42.8** **(*p* = 0.002)**	233.3 ± 50.8	234.7 ± 52.7	229.6 ± 62.5
LV end-systolic volume, ml	177.4 ± 52.7	**139.4 ± 41.3** **(*p* = 0.0001)**	**154.6 ± 48.5** **(*p* = 0.002)**	165.9 ± 51.3	169.3 ± 49.6	168.9 ± 58.2
VO2 peak, ml/kg/min	15.3 ± 3.2	16.5 ± 3.7	**19.9 ± 4.0** **(*p* = 0.006)**	15.8 ± 6.3	15.7 ± 6.7 (*n* = 14)	18.2 ± 6.6
NT-proBNP, pg/ml	891 [473;1181]	**551 [342;771]** **(*p* = 0.004)**	**446 [375;749]** **(*p* = 0.02)**	956.0 [734;1,719]	852.5 [264;1,139] (*n* = 14)	1,075 [519;1,447]
Eq5D (visual analog scale), %	64.6 ± 17.7	**72.3 ± 14.4** **(*p* = 0.03)**	**74.6 ± 14.9** **(*p* = 0.041)**	65.3 ± 17.8	73.1 ± 14.0	**70.3 ± 16.7** **(*p* = 0.01)**
CCM-stimulation, %	98.9[97;99]	99.0[98;99]	99.0[95;99]	98.0[95;99]	98.0[96;99]	96.5[91;99]
Medication, *n* (%)						
ACE-i/ARB	13 (100%)	13 (100%)	13 (100%)	15 (94%)	15 (94%)	15 (94%)
>50% of full dose	8 (62%)	8 (62%)	9 (69%)	10 (67%)	11 (73%)	10 (67%)
Full dose	4 (31%)	5 (38%)	4 (31%)	3 (20%)	6 (40%)	6 (40%)
Beta-blockers	13 (100%)	13 (100%)	13 (100%)	16 (100%)	16 (100%)	16 (100%)
>50% of full dose	8 (62%)	10 (77%)	9 (77%)	11 (69%)	10 (63%)	10 (63%)
Full dose	2 (15%)	2 (15%)	2 (15%)	3 (19%)	5 (31%)	6 (38%)
Aldosterone antagonists	12 (92%)	13 (100%)	13 (100%)	14 (88%)	14 (88%)	16 (100%)
Left ventricular reverse remodeling: comparison of the changes among the study groups at the 6 months, median [Q25; Q75]						
Change in LVEF (absolute), %	4 [2; 11.0]			0.5 [−2; 3]		
	***p* = 0.009**					
*Δ* LV end-diastolic volume, ml	−42.0 [−55; −17]			0.5 [−11.5; 15]		
	***p* = 0.0002**					
Change in LV end-diastolic volume, *Δ* %	17.1 [−20.1; −8.9]			0.25 [−4.7; 5.8]		
	***p* = 0.0002**					
*Δ* LV end-systolic volume, ml	−33.0 [−46; −22]			0 [−7; 10.5]		
	***p* = 0.000001**					
Change in LV end-systolic volume, *Δ* %	−17.1 [−25.5; −15.4]			0 [−5.2; 6.4]		
	***p* < 0.00001**					
Left ventricular reverse remodeling: comparison of the changes among the study groups at the 12 months, median [Q25; Q75]						
Change in LVEF (absolute), *Δ*%	8 [−2; 12]			1 [−3; 4]		
	*р* = 0.2					
*Δ* LV end-diastolic volume, ml	−21 [−40; −16]			−3.5 [−13; 15]		
	***p* = 0.006**					
Change in LV end-diastolic volume, *Δ* %	−9.4 [−19.4; −6.5]			−1.5 [−5.5; 3.8]		
	***p* = 0.02**					
*Δ* LV end-systolic volume, ml	−17 [−24; −13]			0 [−11; 6]		
	***p* = 0.001**					
Change in LV end-systolic volume, *Δ* %	−9.1 [−18.9; −6.5]			0 [−8.7; 3.5]		
	***p* = 0.007**					

ICD, implantable cardioverter defibrillator; FC, functional class; RV, right ventricle; HR, heart rate; SBP, systolic blood pressure; 6MWT, six-minute walk test; LVEF, left ventricular ejection fraction; VO2 peak, maximal oxygen consumption; Δ, the changes.

Bold values indicate that mean values that reach statistical significance.

Abbreviations as in Tables 1 and 2.

* *р* < 0.05, comparison with baseline data in the respective group.

** Data of NYHA functional class have non normal distribution, but for clarity illustration of the indicators dynamics presented as mean ± SD.

**Figure 1:**
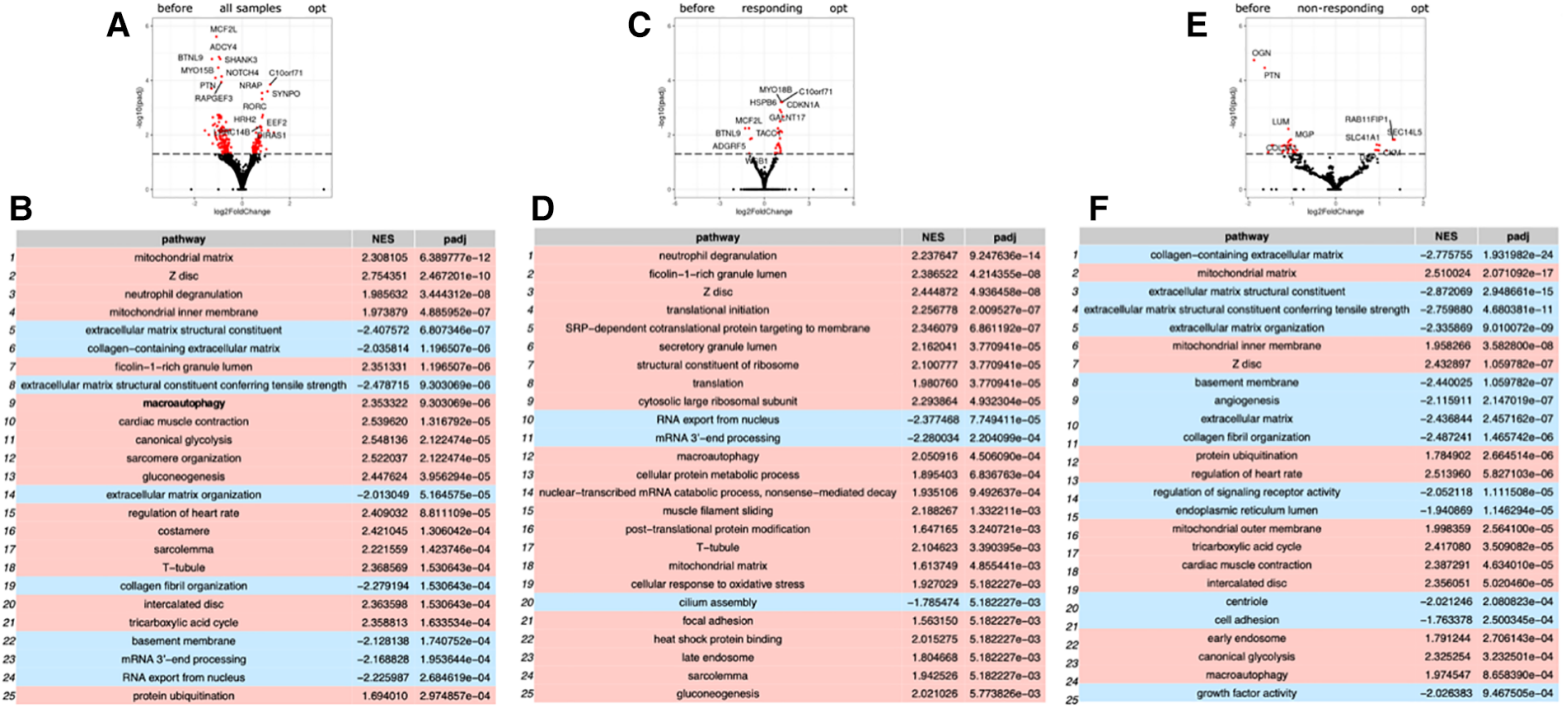
Gene expression analysis by RNA sequencing of biopsy samples from patient before and after CCM treatment. (**A**) Volcano plot illustration RNA-Seq differential expression data. Pairwise comparisons is shown between all samples before and after therapy. (**B**) Gene enrichment analyses for comparison of all before and after CCM therapy samples. (**C**) Volcano plot illustration RNA-Seq differential expression data for pairwise comparisons samples between responders to therapy samples before and after CCM therapy. (**D**) Gene enrichment analyses for comparison of responders samples before and after CCM therapy. (**E**) Volcano plot illustration RNA-Seq differential expression data for pairwise comparisons samples between non-responders to therapy samples before and after CCM therapy. (**F**) Gene enrichment analyses for comparison of non-responders samples before and after CCM therapy.

**Figure 2:**
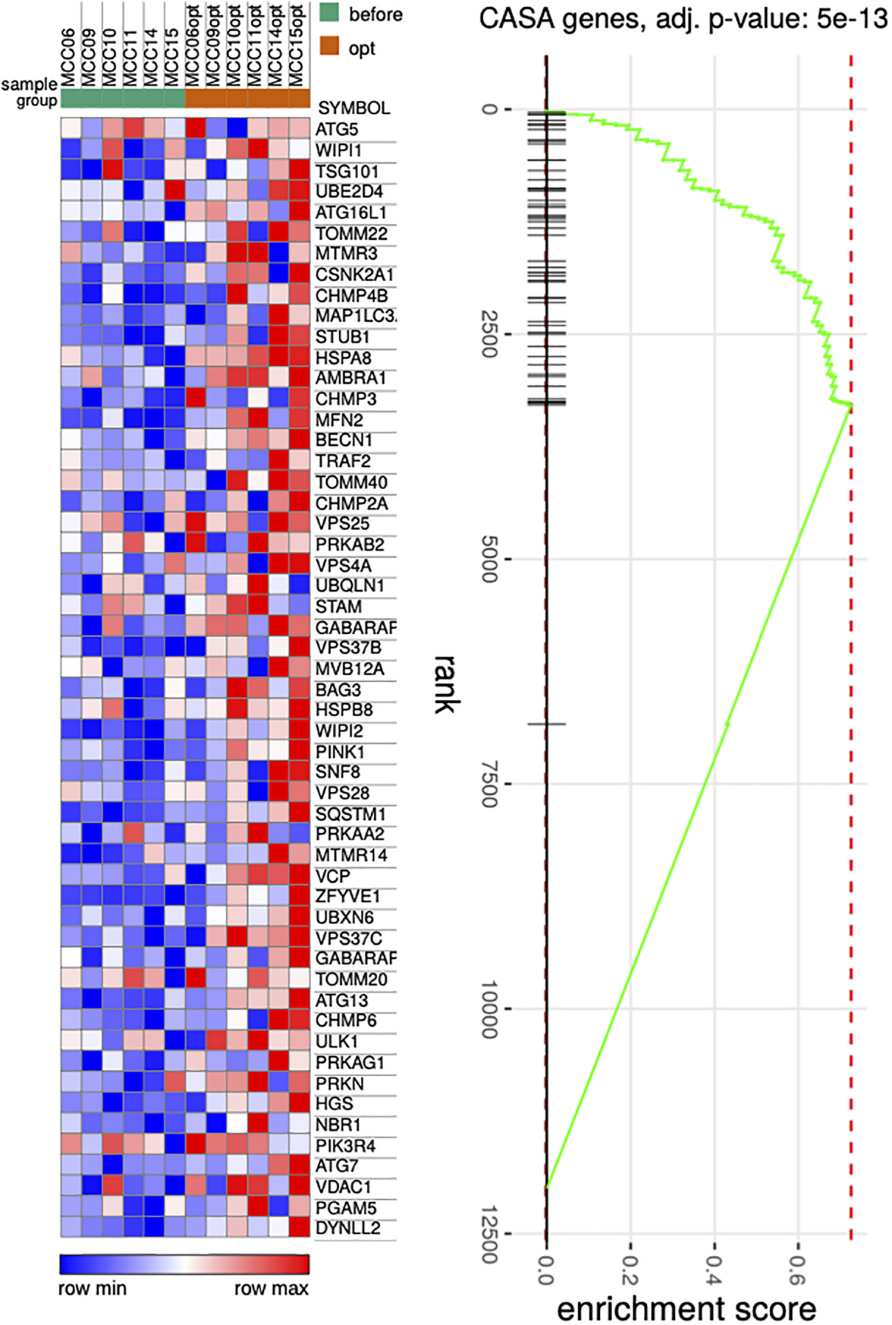
Heat map illustrating genes associated with CASA process. P Pairwise comparisons is shown between all samples before and after therapy. Blue, negative log fold-change (log FC) indicates lower expression; red, positive log FC.

Repeated RNA-seq analysis was performed to identify common transcriptional changes related to CCM as well as to identify transcriptional changes unique to the responder or non-responder groups of patients, the samples were first collected during CCM implantation, and then in patients with later implantable cardioverter-defibrillator (ICD) insertion during the operation. Two consecutive RNA-seq data were collected for 6 patients, including 3 responders and 3 non-responders (Table 1, Supplementary Table S2). The mean time of ICD implantation with repeated myocardial biopsy sampling was 10.6 ± 2.6 months after CCM implantation and did not differ between the responder and non-responder groups. First, we compared transcriptional changes before and after CCM in all the patients. A total of 242 genes were differentially expressed (Figure 1A), with *SYNPO*, *NRAP*, and *RORC* being the most upregulated after CCM therapy and *BTNL9*, *PTN*, *OGN*, *NOTCH4*, *MCF2l*, and *ADCY4* as the most downregulated. Many pathways related to Z-disc structure, mitochondria, and macroautophagy were upregulated in patients after CMM (Figure 1B). A further separate analysis of post CCM samples in responder and non-responder groups revealed that most of the genes upregulated in the entire post-CCM group were represented by the samples from the responder group. We revealed 32 differentially expressed genes, with *HSPB6*, *MYO18B*, *CDKN1A*, *GALNT17*, *KCNK6*, *KCNJ4*, and *SNAP47* being the most upregulated genes; and *BTNL9* and *MCF2l* being the most downregulated genes in CCM responder group (Figure 1C). These upregulated genes included signal recognition particle (SRP)−dependent co-translational protein targeting to membrane, many metabolic genes as well as genes encoding for actin-binding proteins (Figure 1D). Very few genes were upregulated in CCM non-responder group, while several genes (*LUM*, *PTN*, *OGN*) were significantly downregulated, including genes involved in cell adhesion and integrin-complex, collagen and heparin-binding processes (Figures 1E,F**)**. The overall effect of CCM on global gene expression was mainly provided by samples from the responder group and included the upregulation of the genes involved in the maintenance of proteostasis and mitochondrial structure and function. Since macroautophagy was one of the upregulated pathways, we performed a detailed analysis of the genes involved in a muscle-specific type of autophagy—chaperone-assisted selective autophagy (CASA) and mitophagy. The gene set enrichment analysis revealed the significant upregulation (*p* < 5e-13) of the genes involved in CASA and mitophagy in patients after CCM (Figure 2).

**Figure 3:**
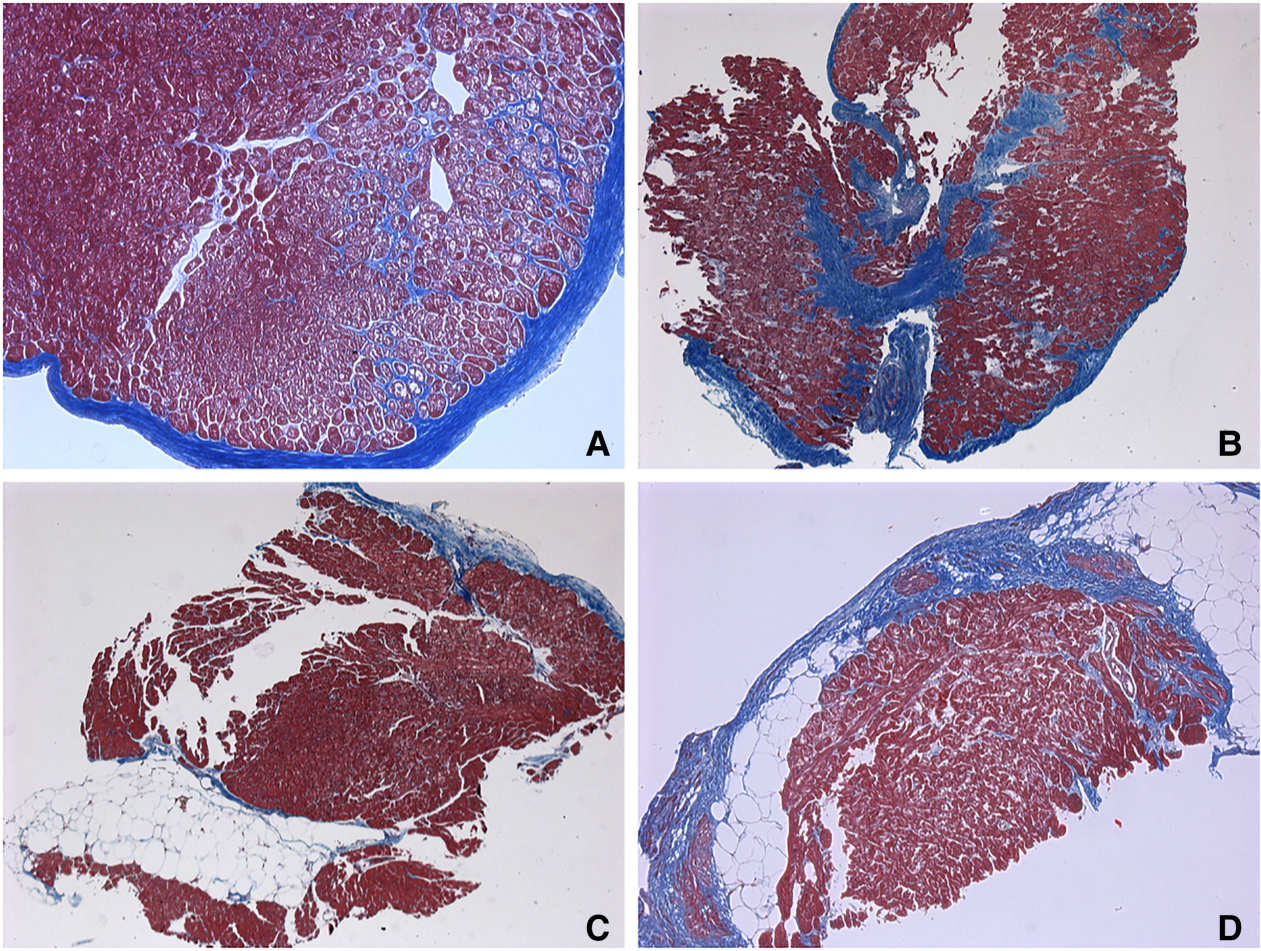
Endomyocardial biopsies. (**A–C**) responders’ samples. (**D**) non-responders’ sample. (**A**) Endocardial fibrosis with a pronounced vacuolar degeneration of cardiomyocytes and fibrosis-braiding of cardiomyocytes. 65 years old, х100. (**B**) Small-focal fibrosis (or Focal fibrosis) with fibrosis-braiding of cardiomyocytes, endocardial fibrosis. 65 years old, ×50. (**C**) Fatty infiltration with endocardial fibrosis. 56 years old, ×50. (**D**) Small-focal fibrosis (Focal fibrosis) with fatty infiltration. 62 years old, х100. Masson's trichrome stain.

To further search the predictive expression markers that could distinguish responders from non-responders based on their baseline characteristics, we compared the baseline transcriptional profile in these groups. The statistical power of this test was lower than in the previous comparison since the number of analyzed samples was less (*n* = 6), and many factors were included into differential expression design (e.g., sex of the donor, batch), which resulted in the almost complete absence of differentially expressed genes (Supplementary Figure S1). We found that many mitochondrial pathways, mitochondrial matrix-related genes, Z disc-protein encoding genes and muscle contraction-related genes were upregulated in responders. In contrast, immune pathways including leukocyte cell-cell adhesion, and neutrophil degranulation were upregulated in non-responders. To exclude the difference in tissue composition, muscle cell cellularity, and the area of fibrosis, the morphological and voltage electrophysiology analysis were performed. We demonstrated that there were no differences in the total cardiomyocyte area and ECG voltage in myocardial samples from responder and non-responder groups (Table 3, Figure 3). Therefore, the observed differences in myocardial expression profile was not linked to different cellular composition.

**Table 3:** Histological analyses and intraoperative parameters of electrodes of 6 patients with repeated endomyocardial biopsy samples (pre- and post-fixation electrode values), mean ± SD.

	Overall (*n* = 6)	Responders (*n* = 3)	Non-responders (*n* = 3)
Percentage of fibrotic areas of endomyocardial biopsy samples, %	13.8 ± 8.6	13.2 ± 8.1	14.0 ± 10.2
Sensed P wave value (RA lead), mV			
RA 0	3.2 ± 0.6	3.4 ± 0.5	3.0 ± 0.8
RA post	4.8 ± 0.4	4.5 ± 0.5	5.0 ± 0
Sensed R wave value (RV lead), mV			
RV 0	10.5 ± 1.2	10.7 ± 0.3	10.5 ± 1.9
RV post	8.2 ± 2.0	9.0 ± 1.0	7.5 ± 2.8
Sensed R wave value (LS lead), mV			
LS 0	9.5 ± 1.1	9.1 ± 1.2	10.5 ± 1.9
LS post	6.6 ± 1.8	7.2 ± 2.0	6.0 ± 1.7

0: at time 0; post: post-fixation; RV, right ventricle; RA, right atrium; LS, lead sensing.

## Discussion

In our study, we demonstrated that 6–12 months of CCM of ischemic HF patients (II-III NYHA) was associated with improvement of clinical parameters and quality of life. By 6 months of CCM, reduction of LVESV ≥ 10% and LVESV ≥ 15% were registered in 45% and 34% patients, respectively. These data are in line with data on 3 months of CCM in patients with HFrEF (III NYHA) (50% with ischemic aetiology) published by Zhang and co-authors who documented the decrease in LVESV ≥ 15% in 39% of cases (9). Of note, in our study, the long-term CCM did not result in significant dynamics of reverse myocardial remodeling in 55% of patients despite improvement in the clinical symptoms and exercise tolerance.

The reverse remodeling of LV under CCM is determined by molecular pathways and previous studies underlined the cardiomyocyte calcium-handling genes as the primary effectors of CCM impact (8, 10–12). However, we did not observe any effect of CCM on either of calcium-related proteins. One of the possible explanations could be the different time point of biopsy sampling in our and previously published studies (12 and 3 months correspondingly). Additional explanation could be the different technical approach utilizing RNA sequencing in a current study compared to previously reported RT-PCR data. Thus, our study provides the information on the later time points of CCM effect and illuminate new and previously unreported genes and pathways linked to a positive effect of CCM. Most of the gene expression effect on CCM raised from the responder cardiac samples leaving the non-responder group as almost “unreactive”. Among the most upregulated are mitochondrial matrix and mitophagy genes, mitochondrial metabolism-related genes, contractile sarcomeric genes as well as structural cardiomyocyte genes. Allover, the CCM-related improvement of myocardial function is associated with expression increase of cardiomyocyte structural and metabolic genes and downregulation of extracellular matrix and collagen synthesis-related genes. This is well in line with data reported by D'Onofrio et al., who demonstrated the improvement of inflammatory circulating biomarkers and markers of fibrosis such as collagen 3, collagen 4, C-cystatin and IL-6 after 6 and 12 months of CCM therapy in patient with LMNA-associated dilated cardiomyopathy (13). However, the observed modulation of gene expression still leaves the question whether the described molecular effects are specifically linked to CCM-related cardiomyocyte changes or simply reflect the cellular processes under positive cardiac remodeling and HFrEF positive dynamics. For example, upregulation of mitochondrial pathways, structural and Z disc-protein encoding genes and muscle contraction-related genes can represent the molecular signature of increased cardiomyocyte contractility and functional myocyte properties as a result of HF treatment independently on CCM. Further experimental and clinical studies with extended control groups and increased number of patients included will allow to answer these questions.

The involvement of several genes linked to CASA and mitophagy prompted deeper analysis of this gene set under CCM. We detected the relative increase in expression of such genes including *BAG3*, *HSPB8*, *HSPA8*, *VSP* and *SQSTM1* as well as *MFN2, VDAC, PINK1 and PRKN* (Figure 2G). CASA has already been reported as essential process degrading damaged components of Z-disc and our data further confirms that positive CASA flux can be one of the attributes of restoration of cardiomyocyte contractile function (14–17).

The increase in CASA-related genes, structural, mitochondrial and contractile genes reflect the late CCM-mediated effects which do not include the direct involvement of Ca-operating genes described as the early CCM effects. Importantly, these effects were mostly represented by responder group samples. This underlines the importance of initial patient stratification and identification of those subjects who have the highest probability to benefit from CCM. We speculate the preservation of mitochondrial structure and metabolism as well as contractile and cytoskeletal apparatus determinate greater effect of CCM. In contrast, the increased expression of immune and inflammatory response genes is associated with moderate or no long-term functional response to CCM. We conclude that the baseline cardiomyocyte status—either more pro-contractile or more proinflammatory—is critical for the long term on cite and remote effects of CCM.

## Study limitations

Due to the very small number of samples available for RNA sequencing the current study represents the pilot project aiming to underline the possible molecular effects of long-term CCM treatment. Further verification and validation of the described tendencies need to increase the number of samples and, possible, to extend the number of time points analyzed. In addition, the data obtained in frame of multicentre study rather than in single center study will allow more accurate and objective clinical data assessment.

## Data Availability

The data presented in the study are deposited in the SRA, NCBI repository, accession number GSE251971. https://www.ncbi.nlm.nih.gov/geo/query/acc.cgi?acc=GSE251971

## Supplementary material

SUPPLEMENTARY FIGURE S1

Gene expression analysis by RNA sequencing of biopsy samples from patient before and after CCM treatment. (A) Volcano plot illustration RNA-seq differential expression data. Pairwise comparisons is shown between all responders and non-responders samples before therapy. (B) Gene enrichment analyses for comparison of all responders and non-responders samples before therapy.
