# Supplementary material for: Molecular effects of cardiac contractility modulation in patients with heart failure of ischemic aetiology uncovered by transcriptome analysis
Source: Front Cardiovasc Med. 2024 Feb 1;11:1321005. doi: 10.3389/fcvm.2024.1321005 (PMC10867213; doi:10.3389/fcvm.2024.1321005)
Supplement: Supplementary file 1 [file Table1.docx]

**Supplemental Table 1 Clinical course and outcome of patients after cardiac ontractility modulation therapy (n=29). Data presented as: n (%), mean±SD, or median [Q25; Q75]**

ICD insertion after the CCM device implantation: 11 (38%)

Device pocket stimulation: 3 (10%)

Leads replacement (4 RV leads): 3 (10%)

HF hospitalizations: 2 (7%)

| **Parameter** | **Baseline** | **6 months** | **12 months** |
| --- | --- | --- | --- |
| NYHA FC, n (%)  I  II  III  NYHA FC, Me [Q25; Q75],  mean ± SD** | 0(0%)  21 (72%)  8 (28%)  2[2;3]  2,3±0,5 | 3 (10%)  26 (90%)  0 (0%)  **2[2;2] ***  1,9±0,3 | 3 (10%)  25 (86%)  1 (4%)  **2[2;2] ***  1,9±0,4 |
| HR, beats/min | 67,1±7,1 | 66,0±7,0 | 65,4±9,4 |
| SBP, mmHg | 114,7±12,6 | 117,2±9,8 | 116,7±14,3 |
| QRS, ms | 115,3±18,3 | 116,7 ± 20,1 | 119,9 ± 21,4 |
| 6MWT, m | 380,3±80,2 | **413,7±71,7 (p=0,004)** | **452,8±74,6 (p<0,00001)** |
| LVEF, % | 25,8±5,7 | **28,7±6,9 (p=0,02)** | **28,7±6,7 (p=0,02)** |
| LV end-diastolic volume, ml | 241,9 ± 53,9 | **223,7±47,2 (p=0,007)** | **226,5±53,8 (p=0,02)** |
| LV end-systolic volume, ml | 171,0 ± 51,3 | **155,8±47,7 (p=0,008)** | 162,5±53,6 |
| VO2 peak, ml/kg/min | 15,8±4,8 | 16,2±5,4 (n=27) | **18,9±5,6 (p=0,001)** |
| NT-proBNP, pg/ml | 902[543;1452] | **616,5[273;1088] (p=0,004)** (n=27) | **749[415;1307] (p=0,025)** |
| Eq5D (visual analog scale), % | 65,0 ±17,7 | **72,8 ±13,9 (p=0,005)** | **72,2 ±15,8 (p=0,003)** |
| CCM-stimulation, % | 98 [96; 99] | 99 [97; 99] | 98 [93; 99] |
| **Left ventricular reverse remodeling: comparison before and after CCM therapy (n=29), median [Q25;Q75]** | | | |
| **Parameter** | | **6 months** | **12 months** |
| Change in LVEF (absolute), % | | 2,0 [-1;5,0] | 2,0 [-2;7,0] |
| ∆ LV end-diastolic volume, ml | | -14,0 [-35; 8] | -14,0 [-35; 1] |
| Change in LV end-diastolic volume, ∆ % | | -6,2 [-16,5; 2,7] | -4,7 [-14,9; 0,6] |
| ∆ LV end-systolic volume, ml | | -14,0 [-31; 0] | -11 [ -18; 0] |
| Change in LV end-systolic volume, ∆ % | | -8,9 [-17,1; 0] | -6,5 [-10,7; 0] |

*p < 0,01 comparison with baseline data in the respective group; **Data of NYHA functional class have non normal distribution, but for clarity illustration of the indicators dynamics presented as mean ± SD; ICD, implantable cardioverter defibrillator; FC, functional class ; RV, right ventricle; HR, heart rate; SBP, systolic blood pressure; 6MWT, six-minute walk test; LVEF, left ventricular ejection fraction; VO2 peak, maximal oxygen consumption ; ∆, the changes.
