# Supplementary material for: Molecular effects of cardiac contractility modulation in patients with heart failure of ischemic aetiology uncovered by transcriptome analysis
Source: Front Cardiovasc Med. 2024 Feb 1;11:1321005. doi: 10.3389/fcvm.2024.1321005 (PMC10867213; doi:10.3389/fcvm.2024.1321005)
Supplement: Supplementary file 2 [file Table2.docx]

**Supplemental Table 2 Parameters of 6 patients with repeated endomyocardial biopsy samples before and after 12-months of CCM therapy**

|  | NYHA, FC | | | LV EDV, ml | | | | LV ESV, ml | | | LVEF, % | | | NT-proBNP, pg/mL | | | VO2 peak, ml/kg/min | | |
| --- | --- | --- | --- | --- | --- | --- | --- | --- | --- | --- | --- | --- | --- | --- | --- | --- | --- | --- | --- |
| months  patient | 0 | 6 | 12 | 0 | | 6 | 12 | 0 | 6 | 12 | 0 | 6 | 12 | 0 | 6 | 12 | 0 | 6 | 12 |
| **HF1** | 2 2 1 | | | 357 | 250 | | 268 | 265 | 164 | 215 | 24 | 27 | 25 | 1137 | 771 | 375 | 16 | 19 | 21 |
| **HF2** | 3 2 2 | | | 280 | 232 | | 240 | 199 | 165 | 176 | 19 | 30 | 28 | 2752 | 2230 | 2689 | 8 | 11 | 14 |
| **HF3** | 2 2 2 | | | 270 | 256 | | 224 | 222 | 167 | 143 | 23 | 33 | 37 | 473 | 416 | 339 | 18 | 22 | 24 |
| **HF4** | 2 2 2 | | | 242 | 251 | | 260 | 162 | 193 | 162 | 27 | 26 | 29 | 902 | 264 | 222 | 17 | 15 | 16 |
| **HF5** | 3 2 2 | | | 241 | 248 | | 232 | 185 | 184 | 188 | 25 | 17 | 19 | 3453 | 2737 | 2152 | 12 | 9 | 9 |
| **HF6** | 2 1 1 | | | 327 | 325 | | 399 | 254 | 252 | 328 | 20 | 20 | 19 | 1764 | 936 | 1769 | 19 | - | 23 |

HF, heart failure patient with serial number; FC, functional class; LV EDV, left ventricular end-diastolic volume; LV ESV, left ventricular end-systolic volume; LVEF, left ventricular ejection fraction; VO2 peak, maximal oxygen consumption
